# Colourful Protection: Challenges and Perspectives of Antibacterial Pigments Extracted from Bacteria for Textile Applications

**DOI:** 10.3390/antibiotics14050520

**Published:** 2025-05-17

**Authors:** Micaela Gomes, Helena P. Felgueiras, Barbara R. Leite, Graça M. B. Soares

**Affiliations:** 1 Centre for Textile Science and Technology (2C2T), Department of Textile Engineering, University of Minho, Campus of Azurém, 4800-058 Guimarães, Portugal; id11181@alunos.uminho.pt (M.G.); gmbs@det.uminho.pt (G.M.B.S.); 2 RDD Textiles, Rua do Arranjinho 381 Fração, Q Pavilhão 17, 4750-803 Barcelos, Portugal; barbara.leite@rddtextiles.pt

**Keywords:** antibacterial activity, bacterial pigments, biocompatibility, green extraction, life cycle assessments, molecular pathways, pigment formation

## Abstract

Bacterial pigments have gained significant attention across multiple industries due to their natural hues and unique functional properties. Beyond coloration, some of these pigments exhibit antibacterial activity, making them particularly valuable in the textile industry as sustainable alternatives to synthetic antimicrobial treatments. Bacteria produce a vast array of pigments through diverse biosynthetic pathways, which reflect their metabolic adaptability and ecological roles. These pathways are influenced by environmental factors such as pH, temperature, and nutrient availability. Key pigments, including carotenoids, melanin, violacein, and prodigiosin, are synthesised through distinct mechanisms, often involving tightly regulated enzymatic reactions. For example, carotenoid biosynthesis relies on isoprenoid precursors, while melanin formation involves the oxidation of aromatic amino acids. Understanding these pathways provides insights into bacterial survival strategies, stress responses, and interactions with their environment. This review examines the dyeing potential of bacterial pigments on natural and synthetic fabrics, highlighting advancements in environmentally friendly extraction methods to minimise the ecological impact. Additionally, it explores safety, biocompatibility, and industrial challenges associated with bacterial pigment applications. Finally, future perspectives on integrating these pigments into various industries are discussed, emphasising their potential as bio-based solutions for sustainable and functional materials.

## 1. Introduction

Bacterial pigments have garnered significant attention in recent years due to their multiple applications in various industries, including food, cosmetics, and pharmaceuticals. These dyes of natural origin offer several advantages over synthetic dyes, such as improved biocompatibility, environmental friendliness, and the potential for multifunctional properties [1,2]. A particularly promising application of bacterial pigments is in the field of textile coloration, where they can not only impart vibrant and unique colours but also provide valuable properties, like antimicrobial activity [3,4].

Textiles, especially those used in healthcare and personal care settings, are susceptible to microbial contamination, which can lead to the development of unpleasant odours, stains, and even the spread of infectious diseases. Conventional textile dyes often lack inherent antimicrobial activity, requiring additional finishing treatments to impart such properties. In contrast, bacterial pigments have been reported to exhibit potent natural antibacterial and antifungal activities, making them attractive candidates for textile applications [3,4,5].

Bacterial pigments offer advantages over other natural dyes due to their independence from specific environmental conditions and their potential for additional beneficial properties [6,7]. These microorganisms are easily genetically manipulated and can be used to colour both natural and synthetic fibres [8,9].

Because of this, bacterial pigments are deemed a sustainable and non-toxic alternative to synthetic dyes for medical textiles. They have antibacterial and antifungal properties, which can improve the functionality and safety of medical textiles [3,7]. By using bacterial pigments in the dyeing processes, the environmental impact of textile production can be reduced while providing effective treatment for patients [10].

This study aims to explore the potential of bacterial pigments with antibacterial properties for application in textile dyeing, especially for medical applications. By leveraging the unique characteristics of these natural dyes, we seek to highlight innovative textile materials and approaches that not only exhibit vibrant and fast colours but also possess inherent antimicrobial functionalities, addressing the growing demand for multifunctional and sustainable textile solutions. The primary objectives of this review are to compile existing information on bacterial pigments and explore their potential applications for antibacterial properties. Additionally, it evaluates and compares the methodologies for the isolation, extraction, and application of these pigments in textiles. This research also provides context for future applications, highlighting opportunities to leverage these properties effectively.

## 2. Literature Review and Data Extraction

A systematic literature search was conducted to identify relevant studies that examine extraction techniques for bacterial pigments, their application to textile substrates, and the study of their antibacterial properties. This search was performed using the following academic databases: Scopus, Google Scholar, and PubMed. It included a combination of the following keywords: “bacterial pigment”, “textile dyeing”, “antibacterial properties”, “pigment extraction”, “life cycle assessment”, and “biocompatibility”. This search was mostly focused on articles published between 2014 and 2024, and only articles published in English were considered.

### 2.1. Inclusion Criteria

Articles were included if they (1) focused on extraction techniques that could be used for the extraction of bacterial pigments and/or their application to textile substrates and/or the analysis of their antibacterial potential and/or their safety to other species; (2) provided experimental data, including ab-based or industrial-scale trials, on the use of bacterial pigments in textiles and/or their characteristics; and (3) were based on empirical research or systematic reviews.

### 2.2. Exclusion Criteria

Studies were excluded if they (1) explored the application of bacterial pigments to non-textile materials; (2) analysed properties other than antibacterial properties, such as antifungal or anticancer; or (3) were purely theoretical or opinion pieces without empirical evidence related to this subject.

### 2.3. Data Extraction

Data extraction was conducted using a table designed to organise key information for the selected studies. The data points included the following:

- Study identification: Authors, publication year, and title.
- Bacterial pigment: The pigment used in the investigation (e.g., prodigiosin) as well as the bacterial species from which it was derived.
- Method: It included extraction techniques as well as the method used for the application to textiles.
- Textile substrate: The type of material used (e.g., cotton, silk, polyamide).
- Results: A summary of the colour fastness results, along with the effectiveness of the bacterial pigment in inhibiting bacterial growth when applicable.

### 2.4. Synthesis of Results

The extracted data were synthesised using a narrative synthesis approach. The studies were grouped by type of pigment and methodology. Key themes were identified, including the species for pigment production and its influence on bacterial growth inhibition and extraction and application techniques and their impact on colour yield.

### 2.5. Limitations of the Methodology

Despite efforts to conduct a comprehensive review, certain limitations must be acknowledged. The focus on English-language articles may have resulted in the exclusion of relevant studies published in other languages. Furthermore, the diversity in methodologies among the included studies posed challenges for a direct comparison of results.

## 3. Molecular Pathways in Pigment Formation and Their Antibacterial Activity

Bacteria produce a vast array of pigments through diverse biosynthetic pathways, reflecting the complexity and adaptability of microbial metabolisms. These pigments serve various ecological and physiological roles, such as protection against oxidative stress, antimicrobial activity, and contributions to pathogenicity. Additionally, their molecular origins and biosynthetic mechanisms can differ significantly depending on the bacterial species and the chemical nature of the pigment itself. Pigment formation in bacteria is highly influenced by external factors such as pH, temperature, and available carbon and nitrogen sources.

Pyocyanin, for example, is a redox phenazine pigment produced by the species *Pseudomonas aeruginosa*. Quorum sensing is the regulatory mechanism that controls pyocyanin production in this species. This system, primarily coordinated by the LasR-LasI and RhlR-RhlI pathways, utilises autoinducers such as acyl-homoserine lactone (AHL) and the *Pseudomonas* quinolone signal (PQS) to upregulate pyocyanin synthesis. Additionally, proteins like MvaT and MvaU enhance PQS activity, further promoting pyocyanin production [11]. The main precursor in pyocyanin biosynthesis is phenazine-1-carboxylic acid. Firstly, it is converted to 5-methylphenazine-1-carboxylic acid betaine by PhzM, an enzyme. This is a methyltransferase that uses S-adenosylmethionine for this process. A methyl group is then added to the nitrogen atom in the phenazine ring. Lastly, PhzS, a FAD-dependent mono-oxygenase, completes the hydroxylative decarboxylation by removing a carboxyl group and adding a hydroxyl group, resulting in pyocyanin formation [12]. Pyocyanin plays a pivotal role in bacterial virulence, contributing to host cell damage through oxidative stress. Pyocyanin exerts its toxic effects by disrupting cellular respiration, inducing reactive oxygen species (ROS) generation, and modulating immune responses [13]. Pyocyanin serves as an alternative electron acceptor. It diverts electrons from NAD(P)H at multiple respiratory chain sites, leading to the accumulation of superoxide anions (O_2_·−) and hydrogen peroxide (H_2_O_2_). This results in oxidative damage to mitochondrial components, including proteins, lipids, and DNA. The oxidative stress triggered by pyocyanin contributes to a loss in mitochondrial membrane potential, which, in turn, activates mitochondrial acid sphingomyelinase (Asm). Asm catalyses the hydrolysis of sphingomyelin to ceramide, a pro-apoptotic lipid molecule that promotes cytochrome c release from mitochondria, ultimately leading to programmed cell death in neutrophils and other immune cells [14].

In bacterial carotenoid biosynthesis, the pathways involve isoprenoid precursors, primarily derived from the mevalonate (MVA) and 2-C-methyl-D-erythritol-4-phosphate (MEP) pathways [12,13]. While the MVA pathway is predominantly found in archaea, fungi, and some bacteria, the MEP pathway is more widespread among bacteria, cyanobacteria, and plant chloroplasts [15]. A crucial step in carotenoid biosynthesis is the formation of geranylgeranyl diphosphate (GGPP), a C20 molecule synthesised by GGPP synthase (GGDPS) through the sequential addition of three IPP units to one DMAPP molecule. Two GGPP molecules then undergo a tail-to-tail condensation reaction to produce phytoene, a colourless precursor that marks the committed step in carotenoid biosynthesis [16]. Liang et al. describe how bacteria synthesise various carotenoids, including C30, C40, C45, and C50 derivatives, using key enzymes like phytoene synthase (CrtB), phytoene desaturase (CrtI), lycopene cyclase (CrtY), and hydroxylases (CrtZ) [15]. C30 carotenoids, such as diaponeurosporene, are synthesised exclusively by certain non-phototrophic bacteria through the condensation of two C15 isoprenoids. C40 carotenoids, including β-carotene and lycopene, are the most common and are produced by many bacteria and fungi. Some Gram-positive bacteria and archaea can further extend these molecules to synthesise C45 and C50 carotenoids, such as decaprenoxanthin, which provide additional stability and photoprotective properties [15]. The regulation of carotenoid biosynthesis is influenced by environmental factors such as temperature, salinity, light, and pH. Stress conditions, including nutrient deprivation, often lead to increased carotenoid accumulation as a cellular defence mechanism [13,14]. Wang et al. researched the effects of high salinity stress (20–100 g/L NaCl) on carotenoid and bacteriochlorophyll production in photosynthetic bacteria. The results demonstrated that photosynthetic bacteria could grow in high-salinity wastewater, and salinity significantly influenced pigment production. Moderate salinity stress enhanced pigment production, while excessive salinity inhibited it. At 50 g/L NaCl, the species showed the highest stimulation, leading to 1.17-fold carotenoid and 1.45-fold bacteriochlorophyll production compared to the control [17]. However, Foong et al. determined that the most effective stress factor for carotenoid production is high light intensity [18]. Carotenoids exert antibacterial effects through a multi-step mechanism that disrupts key structural and functional components of bacterial cells. First, they interact directly with porin proteins on the outer membrane, particularly in Gram-negative bacteria like *E. coli*, leading to the degradation or alteration of these protein channels. This compromises the membrane’s integrity and hampers the transport of nutrients and waste, resulting in the leakage of cellular contents and the loss of homeostasis, which ultimately cause cell death [19]. Additionally, carotenoids increase the overall permeability of the bacterial cell membrane, leading to the uncontrolled loss of vital cytoplasmic components such as ions, ATP, and proteins, further inhibiting bacterial survival [20].

Melanin derives from the transformation of aromatic amino acids, mainly tyrosine [21]. Tyrosine undergoes oxidation to form dehydroxylated diphenol derivatives, which serve as precursors to melanin, and this pathway can diverge in two ways: forming L-DOPA (L-3,4-dihydroxyphenylalanine) with a retained amino group or producing compounds such as homogentisate (2,5-dihydroxyphenylacetate) or homoprotocatechuate (3,4-dihydroxyphenylacetate) without the amino group [22]. These intermediates oxidise either spontaneously or via enzymes, forming dopaquinones or benzoquinones, which then undergo autopolymerisation to form melanin [16,17]. Melanin biosynthesis occurs in Gram-positive and Gram-negative bacteria, including *Streptomyces griseus*, *Bacillus licheniformis*, and *Ralstonia solanacearum* [23]. Supplementing the growth medium with a precursor like L-tyrosine or amino-acid rich proteins, such as peptone and yeast, can be effective ways to achieve higher melanin production in bacteria [24]. Melanin exhibits antibacterial activity through a combination of oxidative, physical, and light-mediated mechanisms. One key pathway involves the generation of reactive oxygen species (ROS), driven by melanin’s redox-active catechol groups, which participate in electron transfer reactions, producing ROS such as superoxide anions and hydrogen peroxide. These ROS inflict oxidative damage on bacterial membranes, proteins, and DNA, ultimately leading to cell death [25]. In addition to oxidative stress, melanin can directly disrupt bacterial cell membranes by interacting with their lipid components, resulting in the leakage of cellular contents and the loss of membrane potential—an effect observed across both Gram-positive and Gram-negative bacteria [26]. Furthermore, a photothermal effect in melanin has been investigated; when exposed to light, particularly in the near-infrared spectrum, it seems to efficiently convert light energy into heat, raising the local temperature to levels that are lethal to bacteria [27].

Prodigiosin, mainly produced by bacteria, is a tripyrrole molecule, meaning it is composed of three pyrrole rings [28]. The biosynthesis of prodigiosin follows a bifurcated pathway, meaning it consists of two distinct branches that produce two key intermediates: Methyl-3-n-Amylpyrrole (MAP) and 4-Methoxy-2,2′-Bipyrrole-5-Carbaldehyde (MBC) [22]. These intermediates are then combined to form the final prodigiosin molecule [22]. MAP is synthesised through a three-step reaction that starts with 2-octenoyl CoA, a fatty acid-derived molecule. Its structure is then transformed in MAP through enzymatic reactions. The second branch of the pathway synthesises MBC, starting from L-proline, an amino acid. Finally, the enzyme PigC catalyses a condensation reaction that links MAP to MBP, forming prodigiosin [21,22,29]. Prodigiosin exhibits antibacterial activity through multiple mechanisms that disrupt the bacterial structure and function. Primarily, it targets the bacterial membrane, integrating into the lipid bilayer due to its hydrophobic nature. This interaction destabilises membrane integrity, causing the leakage of essential intracellular components such as ions, proteins, amino acids, and sugars—particularly in Gram-positive bacteria—without forming discrete pores, but rather through chaotropic disruption [30,31]. Beyond membrane damage, prodigiosin interferes with key cellular processes; it suppresses bacterial respiration, thereby impairing energy production, and inhibits the synthesis of proteins and RNA, which are vital for growth and replication. Additionally, it disrupts cell division, as evidenced by the elongation of treated bacterial cells and the failure to complete cytokinesis, ultimately leading to growth arrest and bacterial death [32].

**Figure 1:**
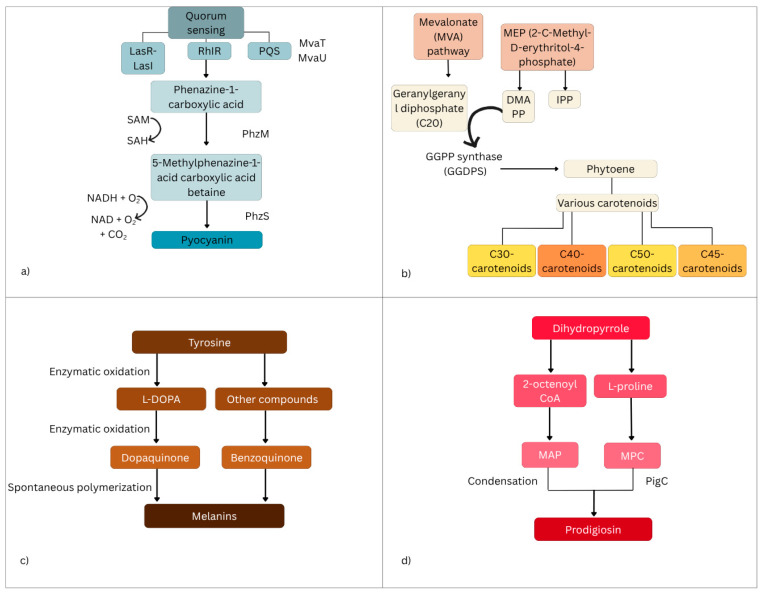
Diagram representation of the molecular pathways for pigment formation in (**a**) pyocyanin, (**b**) carotenoids, (**c**) melanin, and (**d**) prodigiosin.

Figure 1 compares the molecular pathways in the pigment formation of these four pigments.

Understanding the diverse pathways that lead to pigment formation allows us to understand how bacteria adapt to environmental stressors, regulate gene expression, and interact with their surroundings. Many of these pigments play crucial roles in microbial survival, such as protecting against oxidative damage and contributing to pathogenicity. These pathways potentiate the understanding of evolutionary biology, revealing how different bacterial species have independently developed or conserved similar biochemical strategies.

Table 1 summarises the antibacterial mechanisms of the pigments mentioned above, as well as what type of bacteria they are effective against.

## 4. Extraction Methods for Bacterial Pigments

Pigment extraction entails the methodical isolation and separation of a particular pigment compound from its natural source, which, in this case, are bacterial cells [36]. The first step in pigment extraction is cell disruption, which allows the release of intracellular pigments. The pigment can then be extracted by different methods, such as chemical and mechanical techniques [23,24]. Then, to isolate the pure pigment, it can be purified through methods such as (a) chromatography techniques, including high-performance liquid chromatography (HPLC) and thin-layer chromatography (TLC); (b) filtration, using a filter paper; or (c) evaporation, through the use of heat to eliminate the solvent [25,26]. Finally, it can be characterised through the use of analytical techniques, such as UV–visible spectroscopy and Fourier-transform infrared spectroscopy (FTIR), to measure the absorption or reflectance of ultraviolet and visible light in a sample [36].

As interest in sustainable pigment biosynthesis rises, efficient cell disruption methods are crucial—not just for production yield, but also for effective pigment recovery [37].

The scaling up of extraction methods for bacterial pigments can be more economic as well as optimized in order to obtain the most pigment [38,39].

### 4.1. Mechanical Methods

Mechanical extraction techniques have the advantage of being easily scaled up and effective. On the other hand, pigment recovery can be more challenging, resulting in significant debris in the downstream processes [37].

Some examples of mechanical extraction processes are centrifugation, vortexing, filtration, bead milling, high-pressure homogenisation, and ultrasonication. Although there are several mechanical methods for pigment extraction, one of their main disadvantages is the low yielding of the process. This leads to a high amount of pigment in wastewater, which results in higher costs and lower effectiveness [37].

#### 4.1.1. Bead Milling

**Figure 2:**
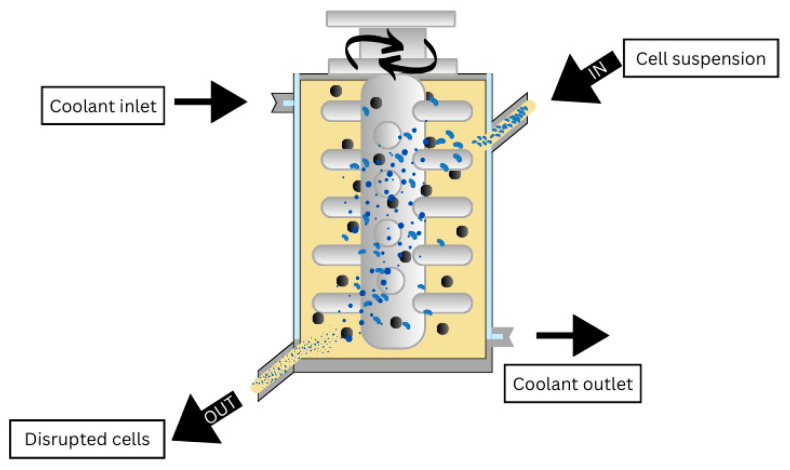
Bead milling scheme.

This process can be operated continuously or discontinuously. It consists of a grinding chamber where the beads are in constant agitation, causing cell disruption [37]. Figure 2 shows the scheme for a bead milling process. Some important parameters to consider for optimising the process are the speed of the agitator, the feeding rate, and the beads’ characteristics, such as size and density [39]. All must be adapted to the characteristics of the microorganism that is being disrupted, as the rigidity of the cell wall can vary [37]. One of the main disadvantages of this process is the amount of energy required [27,28]. For example, this process was used as a pretreatment in the extraction of chlorophyll and carotenoids from the microalgae Chlorella vulgaris in two distinct researches. In one of the researches, glass beads with a 0.4 mm diameter were used [40]. In the second research the milling time spanned from 1 to 60 min, using Y_2_O_3_-stabilised ZrO_2_ grinding beads with diameters ranging from 0.3 to 0.5 mm. The initial cell suspension was placed in a pre-dispersion tank and stirred at 350 rpm to prevent cell sedimentation and ensure the even distribution of solids, and the stirring speed in the grinding chamber was set at 2500 rpm. By applying this method, followed by supercritical carbon dioxide extraction, they were able to increase the process yield by 16% compared to performing the supercritical carbon dioxide method alone [41].

#### 4.1.2. High-Pressure Homogenisation (HPH)

**Figure 3:**
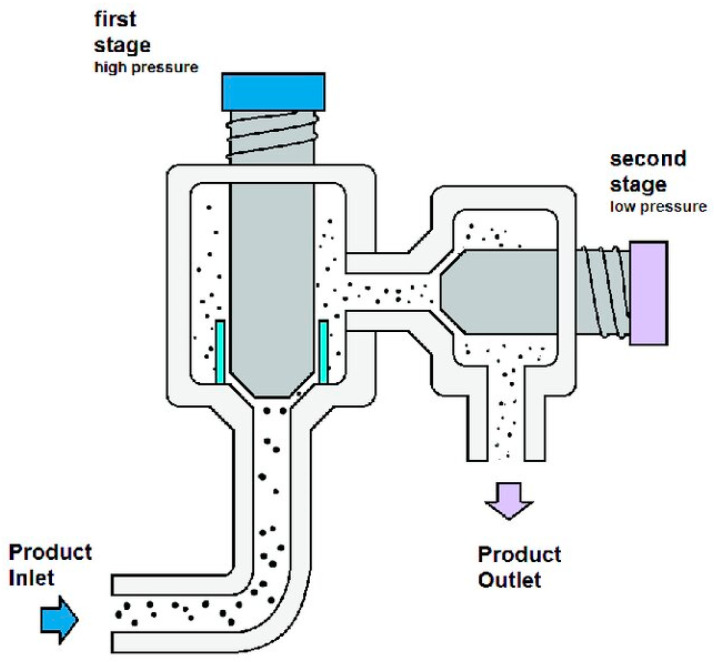
High-pressure homogenisation scheme (adapted from [37]).

This process consists of applying intense pressure to the bacterial suspension, forcing it through a valve. It is then discharged into a chamber with reduced pressure (Figure 3) [37]. Similar to the previous method, the biggest downside of high-pressure homogenisation is the amount of energy required [42]. Additionally, it leads to the creation of cellular debris, which may require extra expenses downstream for the filtration and removal of these by-products while also risking product accumulation in the low-pressure chamber, leading to clumping [37]. A study compared the application of this method with the application of ultrasonication to extract bio-molecules from the microalga *Parachlorella kessleri*. Among these bio-molecules, a non-specified pigment was isolated. The homogenisation pressure was between 400 and 1200 bar, and 500 g of cell suspension was used. The results showed that 1200 bar was the optimal pressure and four passes sufficient for extracting the maximum pigment yield, whereas ultrasonication required 30 min at 400 W to achieve maximal pigment extraction [42].

#### 4.1.3. Ultrasonication

**Figure 4:**
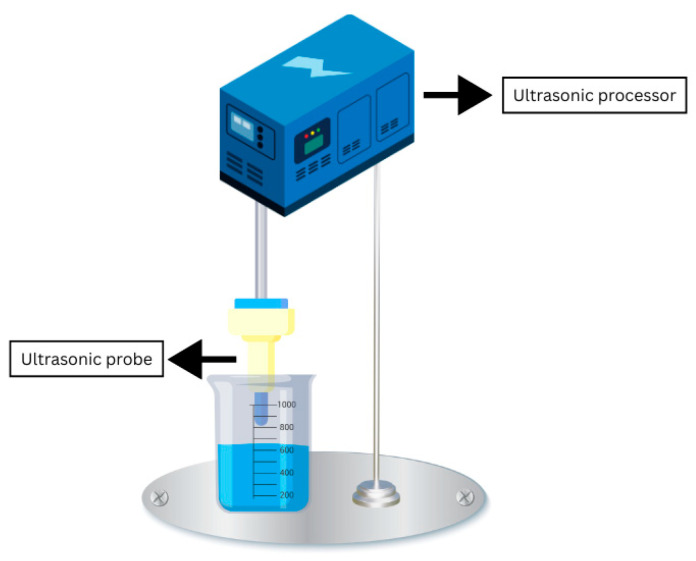
Ultrasonication scheme.

Ultrasonication or ultrasound-assisted extraction (UAE) (Figure 4) can be considered an eco-friendly extraction methodology [43]. It requires the use of ultrasound waves, which are applied to a liquid medium using a resonating rod, resulting in the creation of cavitation. Cavitation happens when vapour bubbles form within the liquid at points where the liquid’s pressure is lower than its vapour pressure. These bubbles expand when subjected to negative pressure and collapse forcefully when exposed to positive pressure, leading to the rapid and intense collapse of the bubbles [37]. This is a low-cost process, but it can cause a lot of deterioration to the compounds [36]. Ultrasound-assisted extraction was used to extract prodigiosin from dried *S. marcescens.* Parameters such as time, temperature, and the solute-to-solvent ratio were optimised to amplify pigment extraction. The best extraction conditions were 17.5 min at 23.4 °C, with a solvent-to-solute ratio of 1:27.2, to obtain an average prodigiosin yield of 4.3 g ± 0.02 g per 100 g of dried cells [44].

### 4.2. Chemical Methods

#### 4.2.1. Organic Solvent Extraction

This method involves the use of organic solvents, such as ethanol, methanol, or acetone, to extract pigments from bacterial cultures [36]. Bacterial biomass is typically harvested and then extracted using a solvent, which should be able to target the pigment, minimising the extraction of undesired compounds [36]. The extraction of carotenes, for example, is typically facilitated using non-polar solvents, whereas xanthophylls are commonly extracted using polar solvents such as acetone or ethanol [45]. Organic solvents with low boiling points are commonly preferred due to their effectiveness in isolating products and enabling efficient recovery or recycling through distillation at low temperatures. This method helps to prevent the degradation of thermally sensitive products. However, it raises concerns related to storage and flammability [46]. Despite its efficacy, organic solvent extraction has drawbacks such as the use of toxic solvents and the potential for environmental contamination. Moreover, this process can be time-consuming, although this can be mitigated by increasing the temperature [47]. For example, the efficiency of carotenoid extraction can be enhanced by conducting the process within a temperature range of 50 to 65 °C since the bacteria wall becomes more fragile. However, temperatures above 70 °C can cause the deterioration of these compounds [47].

Several solvents can be compared for the extraction of a certain compound in order to find the most effective one. For example, the extraction of pink pigment from a species of *Enterobacter* has been carried out with polar solvents as well as non-polar solvents. A study investigated polar solvents such as ethanol, methanol, and acetone as well as the non-polar solvents diethyl ether, hexane, and chloroform. For each one, 15 mL of a 7-day-old culture was first centrifuged at 7800 rpm and 30 °C for 15 min to collect the cell pellet. The resulting pellets were then dissolved in 5 mL of solvent. The final results showed that methanol was the most effective solvent for this specific pigment since it turned pink after cell lysis, making the cell debris white. On the other hand, the other solvents did not gain any pigmentation, making them ineffective for this purpose [48].

Among the available methods, the solvent extraction method using organic solvents such as ethanol and methanol is highlighted as the simplest, most cost-effective, and most used method for extracting intracellular bacterial pigments [49]. However, in the pursuit of sustainability, cutting-edge methodologies often align with green processes. At the forefront of emerging green chemical methods documented in the current literature are the following:

#### 4.2.2. Supercritical Fluid Extraction

Supercritical fluid extraction (SFE) is a method that relies on the unique characteristics of supercritical fluids [50], which have properties resembling both liquids and gases. These fluids, such as supercritical carbon dioxide, possess high densities similar to liquids but exhibit gas-like diffusivity. This combination allows for efficient mass transfer between the extracted solute and the supercritical fluid [51]. The advantages of this process include its simplicity and its duration, which is only between 30 and 60 min [36]. This technique has been applied to extract β-carotene [52] and astaxanthin and lutein [53], which are different types of carotenoid pigments. The first investigation was a success, resulting in an extraction rate of 83%. The team concluded that the success of the process highly depended on (a) the pressure applied during extraction, (b) the temperature, and (c) the flow rate [53]. In a more recent study, the researchers were able to recover 98.6% of astaxanthin and 52.3% of lutein [53]. It is important to note that the higher the cell wall robustness, the harder it can be to disrupt and accomplish a higher extraction yield.

Due to its availability in various industrial activities, such as fermentation and combustion, carbon dioxide serves as an excellent solvent for this technique. Furthermore, it can swiftly transition into a supercritical state, requiring relatively modest temperature and pressure conditions (around 304.12 K and 73.7 bar) [51].

Water can also be used for supercritical fluid extraction. It harnesses the unique properties of water under high-temperature and pressure conditions (100–374 °C, >50 bar) to extract non-polar analytes [54]. This method is less expensive than carbon dioxide supercritical extraction. However, it usually requires further processes to remove the water, such as evaporation [54].

#### 4.2.3. Microwave-Assisted Extraction

This technique requires a small amount of solvent, and the process can be completed in less than 20 min. However, the equipment used is expensive, representing a drawback for this technique [36]. Microwaves have been applied to destroy the cell walls of yeast and, consequently, extract carotenoid and astaxanthin through solvent extraction. The tested conditions were a frequency of 916 or 2450 MHz, an irradiation time between 10 and 500 s, and a microwave output between 50 and 1000 watts. This treatment was followed by solvent extraction with ethanol, methanol, and acetone. The extraction of 4.06 mg of carotenoid per g of yeast and 3.65 mg/g of astaxanthin per g of yeast was accomplished by applying a frequency of 2450 MHz, an output of 500 watts, and an irradiation time of 60 s, followed by solvent extraction for 24 h with ethanol [55].

#### 4.2.4. Enzyme-Assisted Extraction

The enzyme-assisted extraction (EAE) technique employs enzymes such as cellulases, hemicellulases, pectinases, and proteases to break down the cell walls and membranes of bacteria [56]. This degradation facilitates the release and more efficient extraction of intracellular pigments and other compounds. Enzymes generally work under milder pH, temperature, and solvent conditions compared to harsh chemical extraction methods. This characteristic helps to preserve the integrity and activity of the extracted pigments [41,42]. An interesting fact about them is that they can be produced from agro-industrial waste, positively impacting the process and reducing the overall cost of the extraction process [57]. The use of immobilised or recycled enzymes allows their application in multiple extraction cycles. This approach enhances the economic feasibility and sustainability of the process [58]. A study by Alavarsa-Cascales investigated the extraction of anthocyanins from the açaí plant using enzyme-assisted extraction alongside the conventional method of maceration and ultrasound-assisted extraction. The results showed that employing EAE yielded 0.7 mg/g more anthocyanins compared to maceration. Furthermore, when compared to ultrasonication, EAE exhibited a 2.9 mg/g difference, establishing itself as the most effective method among the three techniques [59].

#### 4.2.5. Ionic Liquid-Assisted Extraction

Ionic liquids (ILs) have been used as solvents or co-solvents to extract bacterial pigments and other bioactive compounds [60]. Their low volatility, flammability, and reusability make them potential “green” solvents. IL properties like polarity, viscosity, and toxicity can be tailored by modifying their ion pairs [61]. ILs can selectively dissolve and extract the desired pigments from bacterial cells. This ability arises from their distinct solvation properties and their capacity to interact with specific functional groups [45,46]. This technique can achieve higher extraction yields and purities than conventional solvent-based extraction methods [62]. Moreover, ILs can not only be recycled and reused for multiple extraction cycles, which is crucial from a circular economy perspective [45,46,48], but also be combined with techniques such as microwave-assisted extraction and ultrasound-assisted extraction to further enhance efficiency [61]. However, the initial cost of ILs remains a significant factor compared to conventional solvents [63]. Overall, the process is complex and expensive, requiring careful handling and disposal [64]. As an example of the application of ILs, tetrabutylammonium hydroxide (TBAOH) was used as an ionic liquid to extract microbial melanin from the genus *Streptomyces*. For this process, the supernatant obtained from centrifuging the culture broth was lyophilised into a powder. This powder was then mixed with 1 mL of water and 1 mL of TBAOH. After stirring this mixture, 1 mL of ethyl alcohol was added, followed by centrifugation. The pellet, which contained the pigment, was lyophilised, and the supernatant was used to recover the TBAOH [65].

## 5. Bacterial Pigments in Textile Colouration

Bacterial pigments can be found in nature in a variety of colours, and some of the most common structures are carotenoids, melanin, violacein, and prodigiosin, resulting in a rainbow of colours like cream, brown, yellow, orange, red, purple, blue, and green. Pigments produced by bacteria are secondary metabolites, which usually originate from protective mechanisms against environmental threats, such as external bacteria and fungi, UV light, and other factors of stress for the species [66].

These pigments have been applied in several industries, such as food and cosmetics, and, recently, investigations have been conducted into their application in textiles [6,53]. Bacterial pigments are suitable for textile dyeing since they produce compounds that can generate ionic interactions with textile fibres [67]. These are dependent on different factors, such as the pH conditions of the dyeing bath, a factor that has been demonstrated to hold significance throughout the dyeing process [68].

When dyeing a textile fibre, the goal is to obtain a homogenous colour throughout the material that does not fade when exposed to degrading agents such as light or water. Dye uptake depends on factors such as the type of fibre, the affinity of the pigment, the selected structure for the fabric, the pH, the liquor ratio, and potential pre-treatments that might have been performed on the fabric. When it comes to the dyeing process, this can usually happen through two different methods: continuous dyeing and exhaust dyeing. The first process involves a padding process, where the fabric is immersed in the dyeing bath, padded, dried, and cured until absorption is concluded. The second method is performed in batches and involves the mechanical movement of the fabric or the dyeing bath to promote dye uptake inside the dyeing machine [69]. The dissolution of the dyes is also a crucial step in the dyeing process, and this factor depends on the nature of these compounds. In the case of bacterial pigments, they are usually soluble in ethanol, methanol, water, or acetone [68]. However, more environmentally friendly options are being developed, such as ionic liquids, for example [70].

### 5.1. Carotenoids

**Figure 5:**
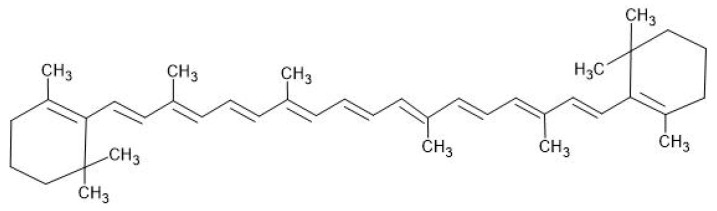
Beta-carotene chemical structure.

In nature, most carotenoid pigments can be found in bacteria, and they usually manifest in red, orange, and yellow tones [71]. Figure 5 represents the structure of beta-carotene, a type of carotenoid. Ram and colleagues identified 20 distinct microorganisms capable of producing carotenoids, emphasising *Flavobacterium* spp., *Agrobacterium* spp., *Micrococcus* spp., *Pseudomonas aeruginosa*, *Serratia marcescens*, *Chormobacterium* spp., *Rheinheimera* spp., and *Arthrobacter* spp. as the predominant species [16]. Figure 4 represents a type of carotenoid, beta-carotene.

Multiple studies have centred on isolating bacteria capable of generating carotenoid pigments. One such study, dating back to 2006, documented the synthesis of carotenoid pigment by two *Escherichia coli* strains after refining the fermentation process. It is suggested that augmenting salt concentrations and refining glucose-feeding profiles could lead to a maximal pigment yield [72]. Dieser and team focused on isolating bacterial strains from Antarctic environments and discovered that carotenoid pigments were sunscreens [73]. In a study released in 2012, the objective was to extract microorganisms capable of generating substantial quantities of carotenoid pigments from various environmental origins. The researchers collected 41 soil samples from distinct areas presenting diverse environmental conditions. Subsequently, they isolated 24 colonies of Gram-positive bacteria exhibiting spherical-shaped and yellow pigmentation. These 24 isolates were subjected to carotenoid extraction using methanol as the solvent. The identification of highly productive carotenoid-producing isolates was confirmed through biochemical characterisation and mannitol salt growth tests. Among the tested isolates, “YCD3b” displayed the highest production of carotenoid pigment, confirmed through spectrophotometric analysis [74].

Another study focused on isolating the pigment-producing bacterium *Lewinella aurantiaca* sp. from surface seawater, which presented orange and yellow colonies that were confirmed to be carotenoid pigments through DNA analysis [75]. Majumdar et al.’s study delved into the utilisation of a bacterium sourced from paper mill effluents to produce a carotenoid pigment. This investigation aimed at employing a “waste treats waste” approach, using paper mill sludge (PMS) as a medium to immobilise *Planococcus* sp. TRC1, an isolated bacterium from wastewater. The study focused on the yellowish-orange pigment produced by this isolate, identifying it as a significant member of the carotenoid pigment family known for its pharmacological importance. Additionally, it explored the antioxidant properties of the pigment. Within a fluidised bed reactor (FBR), the PMS-immobilised bacteria exhibited notable efficiency in removing phenol, lignin, colour, and the chemical oxygen demand (COD) from the effluent following a 60 h treatment. Furthermore, the study highlighted the viability of PMS as an environmentally friendly and cost-effective immobilisation matrix for treating pulp and paper mill effluents. Simultaneously, it underscored the potential for producing a value-added carotenoid pigment from this bacterial isolate [76].

There are also records of the use of bacterial carotenoids in textile dyeing, such as the research by Ahmad and colleagues that applied a yellow pigment derived from *Chryseobacterium* sp. to different fabrics, including natural silk, Dubai silk, linen, Japanese cotton, and Indian cotton. This was achieved by immersing the fabrics in the pigment solution at 80–90 °C. Optimal colour intensity was attained after 60 min of immersion [77].

A study by Bisht and team focused on the application of a red carotenoid pigment extracted from *Rhodonellum psychrophilum* in healthcare, food, and antimicrobial finishes on textiles. To assess the dyeing and finishing behaviour of red pigments, different types of textile materials—cotton, glace cotton, silk, and rayon—were tested. First, red pigment stock solutions (20 mg/mL) dissolved in ethanol were prepared. The fabric pieces were initially immersed in a sodium chloride (NaCl) solution (1% *w*/*v*) for 30 min and subsequently treated with red pigment extracts. They were then subjected to various temperatures (30, 40, 50, 60, and 70 °C) and different dipping times (15, 30, 40, 60, and 80 min). Among the tested fabrics, glace cotton, rayon, and silk did not exhibit significant colouration. However, cotton fabric displayed deep and intense orange colouration when subjected to a temperature of 70 °C for 30 min [78].

In 2021, Ki and colleagues explored the use of bacterial carotenoid pigment as a textile dye. Yellow and orange pigmented bacteria were isolated, cultured, and studied for pigment production under different conditions of pH, temperature, and NaCl concentration. Extracts of carotenoid pigments from these bacteria showed potential as natural dyes for cotton since the process resulted in acceptable colouration (both the yellow and orange pigment remained on the fabric after washing). Here, potassium aluminium sulphate was used as a mordant. UV–visible spectroscopy confirmed their absorbance, suggesting their suitability as eco-friendly dyes in the textile industry [79].

Gurkok focused on extracting an orange pigment from the *Metabacillus idrensis* species and employing it as a dye for cotton fabric in textile applications. The process involved immersing the fabric in the pigment solution at 50 °C for 30 min, followed by mordanting with a thiourea solution at 45 °C for an additional 30 min. Upon washing, the samples displayed a brownish-yellow hue. Although the study did not assess the samples’ fastness or K/S values, the resulting colour appeared visibly vibrant and intense [80].

Overall, bacterial carotenoids seem to have a high affinity for cotton fibres. Most importantly, these dyeing processes were conducted at relatively low temperatures (under 70 °C), which also has an important role in the environmental impact of the dyeing process.

### 5.2. Melanin

**Figure 6:**
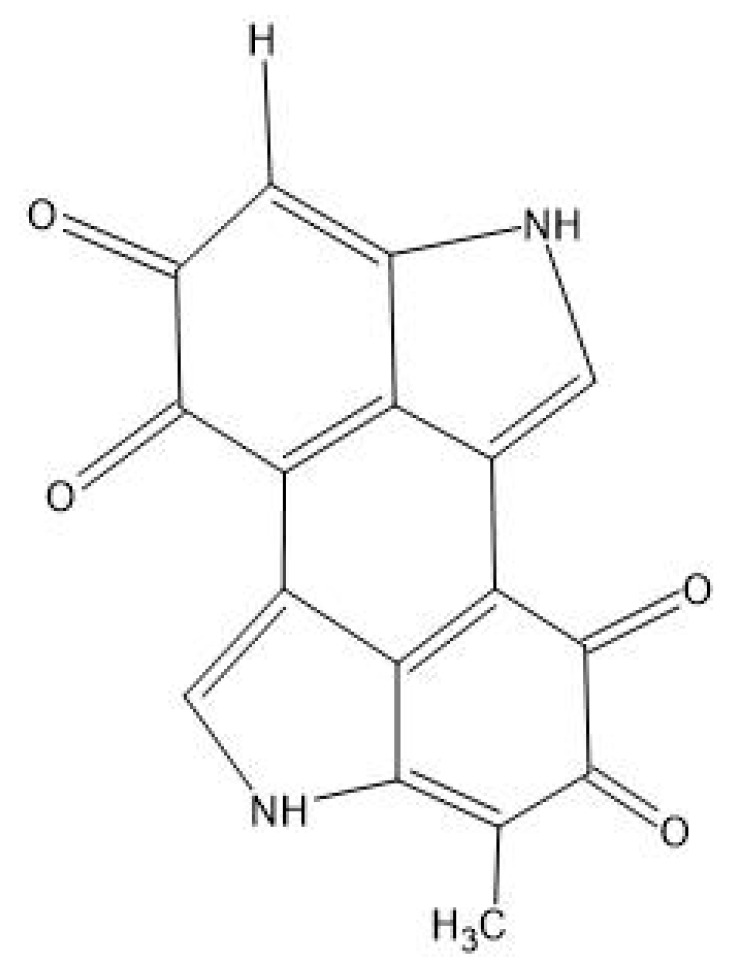
Melanin chemical structure.

Melanins (Figure 6) are usually brown or black pigments, but yellow and red melanins have also been reported [81]. Melanins can be divided into four groups with different structures and diverse functions: eumelanin, which are black to brown melanins; pheomelanin, usually red to yellow; allomelanins, a heterogeneous group that can result in brown and black colours; and pyomelanin, a red–brown type of melanin [82].

Melanins have been extracted through both conventional and modern technologies. Conventional methods include extracellular extraction through acid precipitation and intracellular extraction through alkali extraction, acid precipitation, ultrasonic-assisted extraction, and microwave-assisted extraction, with alkali extraction and acid precipitation being the most common for extraction and purification, respectively. Recent methods include cavitation-based extraction, an eco-friendly method that eliminates the use of toxic solvents. Built on this, other approaches were developed: ultrasound-assisted extraction, negative pressure cavitation extraction, microwave-assisted extraction, and hydrodynamic cavitation extraction [82].

One of the first applications of bacterial melanin as a textile dye dates back to 2011. The team started by selecting the strain *Streptomyces virginae*, which was able to produce a dark-brown pigment. To analyse the dyeing potential of this isolate, it was applied to wool fabrics in dyeing and printing processes. Factors such as pH, time, and medium volume were also tested as influencers on pigment formation. An important finding was that colour strength (K/S) was influenced by pigment formation. The highest K/S was obtained at 50 mL of the *S. virginae* fermented area/250 mL, and it decreased when the medium volume was increased, and pH 6 was ideal for pigment formation. Regarding incubation time, maximum pigment formation was observed after 10 days. The highest level of pigment formation by *S. virginiae* was detected with L-arabinose, followed by glycerol, fructose, mannose, mannitol, and glucose as supplements. Concerning the fastness properties of the dyed and printed samples at optimum conditions, on a scale from 1 to 6 (from the lowest to the highest score, respectively), wash fastness and perspiration fastness varied between 4 and 5, and light fastness reached level 5–6 [83].

In 2021, a team of researchers reported the production of melanin pigment from *Streptomyces glaucescens* and *E. coli* BL21 (DE3). Caffeic acid was introduced into the culture solution to induce melanin production. This melanin was then utilised to dye cotton fibres using six distinct methods. The methods varied, involving the direct dyeing of samples with melanin, the addition of copper ions in the culture solution, the inclusion of copper ions during the dyeing process, the treatment of pigment with laccase enzymes, double dyeing without treatment, and multiple dyeing cycles with a laccase treatment during the process. Notably, the sample obtained from the fourth method, incorporating laccase enzymes, demonstrated the highest fastness for washing, retaining 90.6% of its colour post-washing (level 4–5). In contrast, the sample from the first method retained less than 40% of its original colour after washing, highlighting the efficacy of the laccase treatment in enhancing colour retention [84].

Although the use of bacterial melanin as a textile dye is not common, it seems to have potential for application. This type of pigment can be found in a variety of colours that are not easily found in other groups of pigments, such as brown and black.

### 5.3. Violacein

**Figure 7:**
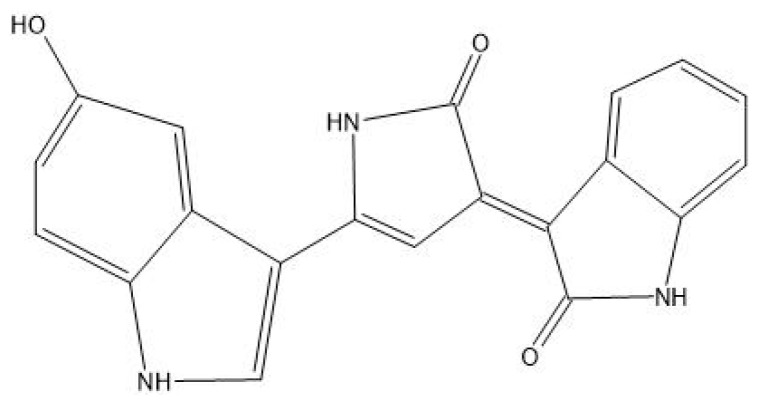
Violacein chemical structure.

This pigment can be found in Gram-negative bacteria, such as *Chromobacterium violaceum* and *Pseudoalteromonas* sp. [66]. Figure 7 represents violacein’s chemical structure.

In a 2000 study, one of the first reports of bacterial violacein being used as a textile dye, researchers explored the utilisation of a pigment isolated from *Janthinobacterium lividum* as a textile dye in various fibres, both natural (silk, cotton, wool) and synthetic (polyester, acrylic, acetate, vinylon, nylon). The investigation began after a silk thread exhibited a blueish-purple hue when wet for an extended period, leading to the discovery of the responsible microorganism. Changing culture conditions, such as medium and temperature, yielded different hues, with a semi-synthetic potato agar medium and 25 °C temperature producing the most pigment. Two dyeing methods were tested, one involving fabric immersion in an extract solution using organic solvents and another where bacterial cells were boiled in water and fabrics dipped. Polyester, acrylic, and rayon resulted in light shades, while nylon was easiest to dye, followed by vinylon, acetate, raw silk, cotton, and wool. Dyeing fastness was evaluated on a staining scale from 1 to 5. Silk exhibited level 1 in light fastness, level 2 in washing fastness, level 3–4 in hot water and acidic sweat fastness, level 3 in alkaline sweat fastness, and level 5 in dry and wet rubbing fastness. Other fibres varied in their fastness properties, ranging from levels 1 to 5 in dry rubbing, wet rubbing, sweat, water, washing, and light fastness [85].

A recent study explored increasing violacein production in *Chromobacterium violaceum* by using formic acid. As tryptophan serves as a substrate for violacein biosynthesis in this species, the study aimed to optimise pigment production by adding formic acid and tryptophan to the culture medium. The results indicated that feeding the bacteria with 0.15 and 0.30 mg/mL of tryptophan yielded the most significant improvements (196% and 185% increases, respectively) in pigment production. Regarding the addition of formic acid, it was observed that concentrations higher than 320 µg/mL led to cell death and ceased pigment production. However, concentrations below this value resulted in increased pigment production as the concentration of formic acid was augmented [86]. A soybean meal medium was compared with commercially available complex growth media to increase violacein production by the same species When employing a 2% *w*/*v* soybean meal (SM2) medium, 496 mg/L of crude violacein was obtained after 48 h of incubation. This amount was 1.62 times higher than that of the crude violacein produced in Luria–Bertani (LB) broth. Moreover, supplementing 100 mg/L of L-tryptophan to 1% and 2% *w*/*v* soybean meal (SMT1 and SMT2) mediums resulted in 1217 mg/L (3.96 times higher than LB) and 1198 mg/L (3.90 times higher than LB) of crude violacein, respectively. Through the optimisation of the culture conditions and L-tryptophan concentration using the Box–Behnken design (BBD) model, up to 1504.5 mg/L of crude violacein was achieved [87].

**Figure 8:**
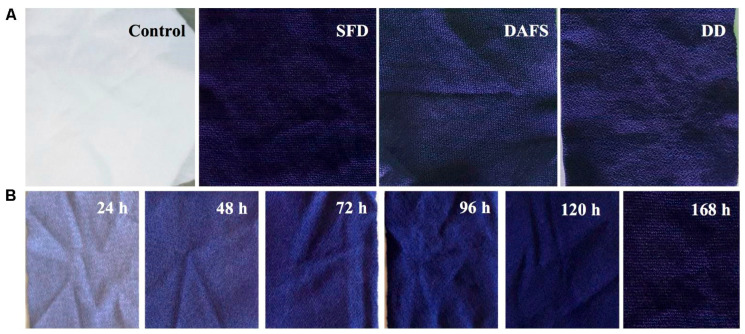
(**A**) Untreated (control) and dyed fabrics via simultaneous fermentation and dyeing (SFD), dyeing after fermentation and sonication (DAFS), and direct dyeing (DD); (**B**) different shades, depending on the incubation time, of purple-dyed polyamide 6 (adapted from [88].)

More recently, a pigment extracted from this species was tested on polyamide 6.6 fabric using three processes (Figure 8). The first process was simultaneous fermentation and dyeing (SFD), the second process was dyeing after fermentation and sonication (DAFS), and the final process was direct dyeing (DD). The most efficient method appeared to be simultaneous fermentation and dyeing, where the fabric was incubated with the microorganism culture under optimal growth conditions. When compared to the other approaches, SFD resulted in a remarkably vibrant purple shade, displaying the highest colour change (ΔE) and the most intense colour (highest K/S). In terms of colour fastness, the pigment showed excellent performance in acid perspiration, alkaline perspiration, and washing tests, achieving a level 5 rating. However, its light fastness was considered poor [88].

A pigment extracted from *Chromobacterium violaceum* was applied to various fabrics, such as pure cotton, pure silk, pure rayon, rayon jacquard, silk satin, cotton, and polyester. Each fabric sample underwent post-mordanting with alum, ferric sulphate, copper sulphate, sodium silicate, and slaked lime, with alum showing the most promising results. It is noteworthy that the natural mordant (slake lime) produced the most vibrant shades for cotton and silk satin, though alum displayed better overall fastness than other mordants. The dyeing process produced a distinct range of blue and violet hues. Among the fabrics tested, cotton and silk satin exhibited superior fastness results, scoring from 1 (for light fastness) to 5 (for perspiration and water fastness) for cotton and from 2 (for light fastness) to 4–5 (for rubbing, perspiration, and water fastness) for silk satin. These findings were later compared with a reactive dye, displaying very similar fastness levels [89]. Similarly, the violet pigment from this species was later extracted for textile dyeing purposes in cotton and silk satin fabrics. The extraction process involved several solvents, such as methanol, acetone, water, and ethyl acetate. For the experiments, the fabrics were tested at 100 °C using different mordants, including ferrous sulphate, sodium silicate, alum, copper sulphate, and calcium hydroxide. Evaluation through K/S and CIELab (L*a*b*) coordinates helped assess colour strength and colour fastness. Water produced the most intense shade, with the lowest L* value achieved on both fabrics. Colour fastness was evaluated on a scale from 1 to 5, and all mordants exhibited satisfactory results, scoring between 3–4 and 5, in washing, rubbing, perspiration, and water fastness tests. For light fastness, copper(II) sulphate (CuSO_4_) showed the best performance, reaching level 2–3 for cotton. However, the highest level achieved in silk satin was level 2, when treated with Ca(OH)_2_ [90].

Unlike most research previously mentioned, silver and titanium dioxide nanoparticles have been added to bacterial violacein. This pioneering method aimed to produce multifunctional textiles with antimicrobial attributes. In contrast to the previously mentioned techniques, the dye was processed into powder form before dyeing. The process involved transferring the pigment solutions to a rotary instrument for 60 min to separate the ethanol from the dye. Viscose fabric was chosen for the study, and due to the significance of physical tests on fabrics and their properties, optical and abrasion stability analyses were carried out on the dyed fabric, resulting in level 2 and 3 ratings, respectively, on a scale from 1 to 5 [91].

Besides all the studies around violacein pigments, there is some scepticism regarding their application at industrial scale, highlighting the gap between the effective and economic feasibility of the process. Despite that, violacein has shown very promising results in textile dyeing, not only on natural fibres but also synthetic fibres. However, from the available literature, it is possible to affirm that further research is needed to find a compatible treatment to improve light fastness [90].

### 5.4. Prodigiosin

**Figure 9:**
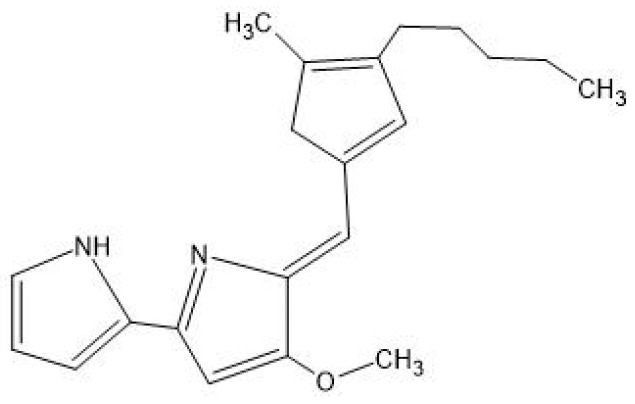
Prodigiosin chemical structure.

Some of the bacteria known to produce prodigiosin (Figure 9) pigments are *Vibrio psychroerythrus*, *Serratia marcescens*, and *Pseudomonas magnesiorubra* [92]. This pigment is known for its red shade and its special properties (antimalarial, antibacterial, and anticancer, for example) [93].

One of the first records of using bacterial prodigiosin as a textile dye was published in 2008, when a team of researchers extracted the pigment from *Vibrio* sp. and applied it to several substrates of synthetic and natural origin. These included wool, nylon, acrylic, and silk. For the dyeing process, a pH of 3.5 was used for wool, silk, and nylon and a pH of 4.5 was used for acrylic. Testing was conducted at 80 °C for 60 min. The study revealed that the pigment was not very stable at high temperatures, similar to other natural dyes [94].

Ramesh and colleagues sought to analyse the array of marine pigmented bacteria in India, leading to the discovery of the red-pigmented bacteria *Zooshikella* sp. and *Streptomyces* sp. Among the 180 samples collected, 14 distinct pigmented species were identified. Following this discovery, two particular species exhibited vibrant red colouration, showing promise for potential applications as colourants [95].

Red biochrome was isolated from *Serratia* sp. and tested on cotton and wool as a natural dye. They were able to achieve a high colour yield (K/S value) on both fabrics. Optimal dye absorption was achieved by dyeing the samples at 50 °C for 50 min, utilising a dye concentration of 4.3 g/L and maintaining a bath ratio of 1:20 (*w*/*v*). Through mordant treatments, acceptable washing and light fastness were achieved, indicated by the colour change rate: 18.1 K/S for cotton and 5.9 K/S for wool in terms of washing fastness, and 13.4 K/S for cotton and 4.4 K/S for wool regarding light fastness [96].

*S. marcescens* was used to dye wool, cotton, silk, nylon, and polyester, all fibres in the same colour and intensity. Fastness results showed that the pigment was stable in acidic (pH 3) and neutral conditions but not in basic pH (greater than 13). It also showed stability when exposed to bleach and high temperatures (100 °C), but it was not resistant to light [97]. Similarly, a prodigiosin pigment from the same strain of *S. marcescens* was used for textile dyeing. However, in this study, they only selected substrates from natural fibres, namely, raw cotton, pure silk, and China silk. The dyeing parameters were also tested, with temperatures varying from 20 to 90 °C, pigment concentrations varying between 2 and 14% owl, a pH between 4 and 9, a retention time between 20 and 120 min, and, finally, salt concentrations maintained at 1 g/L. They concluded that 100 min was the optimal time, while the optimal temperature varied for each fibre (70 °C for pure silk and 60 °C for both other fibres). The 5% owl was determined as the optimal concentration, and the pH that allowed maximum pigment exhaustion was 6. When it comes to fastness, pure silk obtained the best results, with level 4-5 for washing, rubbing, and light fastness when evaluated on a scale from 1 to 5 [98].

Twenty-three isolates of marine samples were classified as producers of prodigiosin and used to dye synthetic and natural fibres. From these, two pigments, from *Serratia rubidaea* and *S. marcescens*, were selected for subsequent analysis and extracted using two solvents, methanol and acetone, with acetone showing the best results (50.4 μg/10 mg biomass). In the dyeing process, two distinct mordants were also tested, ferrous sulphate (FeSO_4_) and aluminium sulphate (AL_2_(SO_4_)_3_), in pre-, simultaneous, and post-mordanting. Simultaneous mordanting with FeSO_4_ was the more effective method, resulting in the highest colour yield for wool and nylon [99].

A study aimed at developing an eco-friendly approach for extracting prodigiosins via liquid fermentation and applying them to acrylic fabric was conducted using pigments from *S. marcenscens* and *Staphylococcus aureus*. Different surfactant fermentation systems were employed to extract pigments from within the thalli to the exterior. The pigments were then applied to the fabric at a high concentration using an infrared dyeing machine, maintaining a bath ratio of 1:20 (*w*/*v*). Temperature analysis revealed that temperatures below 80 °C hindered the red dye’s penetration into the fibre, with the most intense hue achieved at 100 °C. The study meticulously examined the dye bath’s pH, determining that higher pH levels softened the colour depth, with an optimal pH of 2.8 being established for maximum intensity. The research found that non-ionic surfactants, particularly Tween 80 at an 18 g/L concentration, were more effective than anionic surfactants, achieving a maximum transfer ratio of 94.3%. This novel method facilitated the efficient extraction of prodigiosins without the use of organic solvents. Moreover, a cationic dyeing technology based on a nano-suspension system was developed, imparting deep colour and antibacterial functionality to the acrylic fabric [100].

Another study used *S. rubidaea*, found in bivalves, to extract the pigment and dye various cotton (linen, baft, gabardine, jersey) and synthetic fabrics (chiffon, satin, Dacron, polyester). The dyeing process was carried out using 40 mL of the extract at 80–90 °C for one hour, and then the fabrics underwent post-mordanting with ferrous sulphate (FeSO_4_), copper(II) sulphate (CuSO_4_), sodium bicarbonate (NaHCO_3_), and lemon. The choice and concentration of mordants had a significant impact on the final hue of the dyed fabric. Copper sulphate was identified as the most effective mordant for its fastness results when applied at 60 °C for 20 min. Synthetic fabrics exhibited a higher affinity for the prodigiosin dye than cotton fabrics, mostly due to the inability to form hydrogen bonds between the dye and the cellulose. Light fastness showed “very good” results for satin and chiffon (both synthetic fabrics) but “poor” for linen, baft, gabardine, Dacron, polyester, and jersey. However, the washing fastness results were rated “excellent” for all samples, suggesting that prodigiosin, as a natural pigment, is less stable in light than synthetic pigments [9].

Prodigiosin is one of the most frequently utilised bacterial pigments in textile applications [101]. It has exhibited promising results across a wide range of substrates, encompassing both natural and synthetic fibres. Moreover, it is prevalent among various species. Despite its somewhat restricted palette of colours, its satisfactory colourfastness and widespread availability bestow a significant advantage over other types of pigments.

Table 2 compiles all the applications in textiles that were mentioned.

## 6. The Antimicrobial Properties of Bacterial Pigments for Textile Functionalisation

Bacterial pigments like prodigiosin and pyocyanin can disrupt the cell membranes of both Gram-positive and Gram-negative bacteria, leading to leakage of the cellular contents and cell death [38]. The lipophilic nature of pigments allows them to permeate the bacterial cell membrane, altering its permeability and integrity [102]. Lately, studies have focused on the possibility of using these pigments as innovative approaches to achieve antibacterial finishes on textile substrates.

A prodigiosin pigment (red) was extracted from *S. marcescens* and subsequently applied to acrylic fabric to obtain antimicrobial textiles. Remarkably, the dyed acrylic fabric exhibited significant antibacterial efficacy, demonstrating a substantial 81.8% antibacterial rate against *S. aureus*. Additionally, it hindered the cellular metabolism and division of *E. coli* [101]. Later, the authors applied this pigment to lyocell. This time, they prepared a nano-suspension of the pigment, and the dyeing process was carried out for 20 min at 90 °C, with a pH of 3. An anionic modification of the fibres was also performed by introducing various concentrations of 1,2,3,4-butanetetracarboxylic acid (BTCA) and sodium hypophosphite into the deionised water and thoroughly mixing the components to create modified solutions. The results showed that the treated fabrics without this modification had a better bacteriostatic effect against *S. aureus* (>99.9%). However, the treatment allowed for more intense shades when compared to the ones without treatment [103].

Similarly, pigments from *S. marcescens* were applied to cotton to achieve an antimicrobial finish on the substrate. The refinement of growth conditions unveiled that utilising a yeast malt extract medium at a pH of 7, maintaining a temperature of 24 °C, and allowing for a growth duration of 3 days resulted in the highest yield of prodigiosin. In this case, the dyeing process was described as a coating and was conducted for 48 h, during which the samples were coated with 30 mL of the prodigiosin extract. The red coloured samples were tested against several pathogens by the well diffusion method, emphasising the activity against *S. aureus* and presenting a zone of inhibition of 38 mm [104].

The application of bacterial pigments in textiles to ascertain whether the antibacterial properties of these pigments would endure after undergoing this process was assessed for the red pigment derived from the marine bacteria *S. rubidaea*. The test was performed on different fabrics, including cotton and synthetic materials (chiffon, satin, Dacron, and polyester). The synthetic fabrics demonstrated a greater affinity for this pigment, and chiffon displayed the most promising outcomes when tested against pathogens, namely, a 95% reduction in *E. coli* and a 97% reduction in *S. aureus*. In linen, a 97% reduction against E. coli could be observed as well as a 70% reduction against *S. aureus*. However, for gabardine, only a 19% reduction against *E. coli* and a 15% reduction against *S. aureus* were reported [9].

Twenty soil samples from various locations were collected, and pigment-producing bacteria were isolated and identified. Using 95% methanol as the solvent, the pigments from these isolates were extracted. Among the 13 pigmented bacterial strains, 4 (named S4O, S11Y, S14P, and S17G) showed significant pigment production on nutrient agar, prompting further investigation. Through analysis, the extracted pigments were identified as carotenoids (S4O, S11Y, and S14P) and pyocyanin (S17G). Antibacterial testing highlighted considerable activity against S. aureus in all four pigment extracts, with the green pigment from isolate S17G demonstrating the highest efficacy against both Gram-positive and Gram-negative bacteria [105].

A blue–green hue extracted from *P. aeruginosa* was used to dye silk, wool, cotton, and polyester, with wool exhibiting the greatest affinity (64.54%). In terms of antibacterial efficacy, the diameters of the zone of inhibition were measured, where it exhibited the most pronounced inhibition against *S. aureus* (11.5 mm in silk). The effect was also successful against *E. coli*, *Bacillus subtilis*, and *Salmonella typhi* [106]. In contrast to the findings of previous studies, the results of this research challenge the assumption that substrates displaying greater dye uptake inherently demonstrate superior effectiveness in terms of antibacterial properties.

*Streptomyces* species are widely studied for their pigments with antimicrobial properties. A pigment from *Streptomyces cyaneofuscatus* was used to dye silk and cotton. Silk was identified as the substrate with the highest affinity for this pigment due to its saturated orange shade, and its activity against *S. aureus* was tested and evaluated as 95% [107].

A yellow bioactive pigment derived from a different *Streptomyces* species, *Streptomyces thinghirensis*, was extracted to harness its antimicrobial properties. The bacteria were isolated from the plant rhizosphere, followed by pigment production in a maltose casein medium, optimised through fermentation. Subsequently, this pigment was applied to two substrates, cotton and polyester, to generate antibacterial textiles. Stability tests indicated a greater affinity for polyester over cotton, and both textiles were assessed for their efficacy against *S. aureus*, showing promising antibacterial effects [108].

Pallath and colleagues were involved in the extraction of a prodigiosin pigment from *S. marcescens*, MBM-17. The team conducted experiments to determine the optimal growth conditions for maximum pigment production, identifying pH 4, 37 °C, and 72 h in a nutrient agar medium as yielding 15.56 g/L of pigment. Testing the pigment against six organisms showed the highest zone of inhibition against *Bacillus* sp. (16 mm), followed by *Salmonella* sp. (13 mm), *S. aureus*, *E. coli* (11 mm), *Klebsiella* sp. (10 mm), and *Pseudomonas* sp. (9 mm) [109].

Similar results were obtained when a red pigment, also from S. marcescens, was extracted and studied for its structure along with its antimicrobial and antioxidant activities. The antimicrobial effects of the pigment were examined against various pathogenic microbes, including *S. aureus*, *B. subtilus*, *E. coli*, *P. aeruginosa*, *Candida albicans*, and *Aspergillus niger*. The pigment exhibited the highest inhibition against *C. albicans* (24.34 ± 2.47 mm), while comparable inhibitory effects were observed for the other pathogens: *E. coli* (19.34 ± 1.67 mm), *B. subtilus* (20.34 ± 2.17 mm), *S. aureus* (20.67 ± 2.56 mm), and *P. aeruginosa* (21.47 ± 1.86 mm) [110].

The antimicrobial activity of 12 crude cell extracts from *Streptomyces* strains was investigated against four microorganisms: *K. pneumoniae*, *P. aeruginosa*, *Staphylococcus epidermidis*, and *C. albicans*. Growth inhibition zones were only evident against Gram-positive *S. epidermidis* and yeast *C. albicans*. All extracts displayed varying degrees of antibacterial activity against the tested *S. epidermidis*, ranging between 4 mm and 6 mm. The dyeing process involved incubating the samples in a growth medium with pigment-producing cultures using multifibre strips conducted under both dynamic and static conditions. Generally, dynamic dyeing showed notably more intense results, with polyamide, silk, and wool exhibiting the deepest shades. Subsequently, these two pigments were applied to polyamide and polyamide-elastane knits and incubated with the fabrics for 3, 5, and 7 days. The 7-day incubation period demonstrated the most promising outcomes, achieving a K/S value of 9 for polyamide-elastane and a K/S value of 8 when applied to polyamide [111].

## 7. Safety and Efficacy of Bacterial Pigments in Textiles

Examining the safety and effectiveness of bacterial pigments in textile materials is paramount, especially considering their potential as natural colourants. This evaluation is crucial for ensuring consumer well-being and environmental preservation. Given the increasing demand for eco-conscious processes and biocompatible materials, for specific applications, it is vital to assess the compatibility of bacterial pigments.

Bacterial pigments have gained attention for their potential biomedical applications [112]. Biocompatibility refers to the ability of a material or substance to interact with living organisms, particularly without causing harm or damage [113]. In the context of human cell lines, biocompatibility implies that the material or substance being examined does not induce adverse reactions or toxic effects upon contact with human cells. Instead, it should support normal cellular functions and behaviours without causing harm or disruption [113]. These pigments, which are produced by a variety of bacteria, are environmentally friendly and biodegradable [114] while also exhibiting antimicrobial and antioxidant properties, further enhancing their potential for medical and industrial use [1]. A study focused on biocompatibility with the human cell line of a melanin pigment from Dietzia schimae concluded that, at a concentration of up to 250 μg/mL, the melanin was not toxic towards human fibroblast cells [2].

Overall, biocompatibility is a critical consideration in the design and development of materials for biomedical applications, including implants, medical devices, drug delivery systems, and tissue engineering scaffolds.

**Figure 10:**
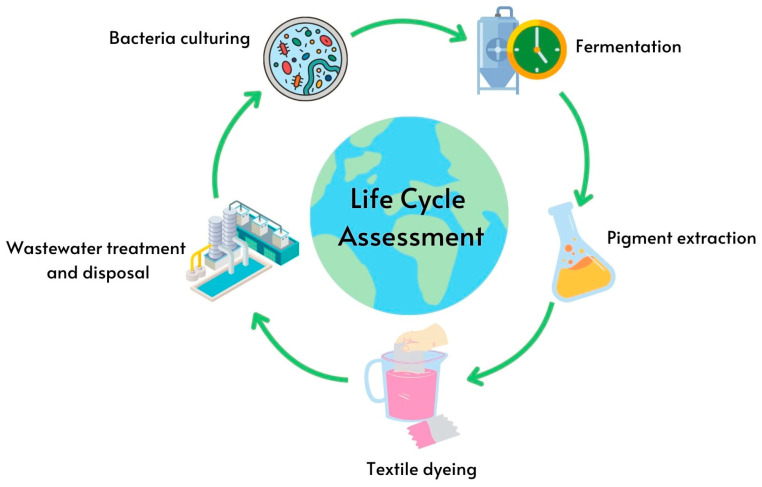
Life cycle assessment of a bacterial pigment.

Life cycle assessment (LCA) is defined as the systematic analysis of the potential environmental impacts of products or services during their entire life cycle, from raw material extraction and production to distribution, use, and end-of-life disposal or recycling [115]. Figure 10 expresses a complete LCA of a bacterial pigment applied to a textile substrate. The LCA of bacterial pigments is a complex process that involves the analysis of various pigments at different stages of growth. Significant differences were found in the pigments of cyanobacterial strains at different incubation periods (7, 14, 21, and 28 days), with maximum content observed at 7 days of incubation [116]. Another study had the purpose of identifying hotspots in pigment production using *Cyanobium* sp., with the intention of achieving the most sustainable scale-up process. This research highlighted cultivation as the predominant source of environmental impacts, largely driven by high electricity demands. While lab-scale processes showed heavy reliance on energy-intensive equipment like freeze dryers, demonstration and industrial scales benefited from process optimisations, such as open-air systems and more efficient drying techniques. Sensitivity analyses revealed that strategic changes—particularly reducing electricity use, adopting renewable energy mixes, and implementing ozone sterilisation—can significantly lessen environmental burdens. The researchers proposed that the stages of reactor cleaning, culture medium sterilisation, and biomass drying should be the focus when trying to reduce the impact. They estimated a reduction of 85% in the impact of the production and extraction processes by implementing the suggested changes.

An LCA study was conducted to study the production of pigments from the macroalga *Saccharina latissimi*. The process used for the extraction was an ionic liquid + oil + water system, composed of 84% of an aqueous solution of a tensioactive phosphonium-based ionic liquid and 16% sunflower oil. They determined the environmental impact of this process and compared it to the possibility of reusing the ionic liquid. The results showed this change could decrease the carbon dioxide impact from 35.5 g CO_2_ to 31.4 [117].

Comparative LCAs of bacterial and synthetic pigments are also crucial to understand the true differences between them. While these studies are limited, insights from related studies on other natural pigments and synthetic counterparts can be helpful.

A study was conducted in Norway to compare the environmental impact of microalgal pigments and synthetic pigments. A sensitivity analysis was performed to compare the environmental impacts of using hydropower with Norway’s field mix for producing the natural pigment. The LCA results were presented based on the impact categories outlined by the Product Environmental Footprint Category Rules (PEFCR). The total single score for the natural pigment was 11.7 mPt, with sodium nitrate and electricity being the main contributors to its environmental impact. When using the country mix for electricity, the sensitivity analysis showed an increased score of 18.2 mPt. In comparison, the synthetic pigment had a lower total score of 0.824 mPt, with methanol being the primary source of its environmental impact. The sensitivity analysis indicated that selecting a different electricity source can lower the environmental impact [118].

Another comparative study focused on astaxanthin production through bacterial, algal, and synthetic approaches. In addition to comparing the environmental impacts of the three production methods, the researchers conducted a sensitivity analysis that was focused on the source of energy. Chemically synthesised astaxanthin exhibited a lower environmental impact across nearly all assessed categories, except for ozone layer depletion, than natural astaxanthin produced via bioconversion. Although bacterial bioconversion using *Corynebacterium glutamicum* and aquaculture side streams demonstrated higher energy consumption than the algal process, it showed significantly lower impacts in key categories, such as abiotic depletion, acidification, human toxicity, ozone depletion, and photochemical oxidant formation. Importantly, upscaling bioconversion from laboratory to pilot scale markedly reduces environmental burdens, and the choice of renewable energy sources, such as hydropower, can further lower impacts by up to 96.9% for bacterial production. Consequently, after energy source optimisation, natural astaxanthin production could achieve an environmental performance comparable to that of chemical synthesis. The fed-batch fermentation step was identified as the primary environmental hotspot, suggesting that targeted optimisation of this stage could further enhance the sustainability of bacterial astaxanthin production [119].

Performing a comprehensive LCA for bacterial pigments is instrumental in elucidating their environmental footprint and sustainability throughout the entire production chain. This assessment spans from the initial discovery and cultivation of pigment-producing microbes to the optimisation of production techniques and the subsequent purification and formulation of the final pigment products. Such insights are indispensable for devising strategies aimed at enhancing the commercial viability and scalability of bacterial pigments as eco-friendly alternatives to synthetic counterparts. By conducting an LCA, it becomes possible to scrutinise aspects like the carbon footprint and energy consumption associated with different production methods, the biodegradability and recyclability of bacterial pigments compared to synthetic alternatives, the feasibility of utilising renewable feedstocks and by-products, the efficiency and environmental repercussions of downstream processing and purification procedures, and the overarching environmental and economic sustainability of bacterial pigments as a whole.

## 8. Challenges Associated with Bacterial Pigment Production and Application

The use of bacterial pigments presents several challenges that must be carefully navigated to realise their full potential in several applications. From issues related to pigment extraction and purification methods to considerations of pigment stability, compatibility with different substrates, and scalability of production, each aspect presents its own set of challenges [120].Additionally, concerns regarding regulatory compliance, safety, and environmental impact further underscore the multifaceted nature of these challenges [121].

Scaling up bacterial pigment production to meet the substantial global market demand, which is presently unmet, is imperative. In addition, it is essential to enhance production yields and implementation and lower costs to fulfil market requirements [105,106]. A major factor is the reliance on costly raw materials, including synthetic growth media and substrates, which makes microbial pigment production more expensive. For instance, producing microbial β-carotene can cost around $1000 per kilogram, whereas its synthetic equivalent costs approximately $500 per kilogram [122,123]. The extraction phase is often labour-intensive and demands the use of expensive solvents and specialised equipment, sometimes contributing to over half of the total production expenses [120].

Navigating the existing legislation and regulations on the use of microbial pigments in food, pharmaceuticals, and cosmetics presents considerable challenges. Obtaining regulatory approval and fostering consumer acceptance of native pigment-producing microbes is essential [25,106]. Bacterial pigments intended for use in food, pharmaceuticals, or cosmetics must navigate strict regulatory frameworks to ensure consumer safety. In both the United States and the European Union, these pigments are subject to comprehensive safety evaluations assessing toxicity, allergenicity, and purity. In the U.S., any pigment used as a food additive must receive pre-market approval from the Food and Drug Administration (FDA), under the Food, Drug, and Cosmetic Act, unless it qualifies as “generally recognized as safe” (GRAS) by experts. Similarly, in the EU, the European Food Safety Authority (EFSA) evaluates food additives for safety prior to approval. Furthermore, if bacterial pigments exhibit antimicrobial properties, they may be classified as biocidal products under Regulation (EU) No 528/2012. This designation requires an additional regulatory pathway, including the authorisation of active substances and any treated articles. These layered regulatory requirements underscore the importance of robust safety data and clearly defined pigment functionality when seeking market approval [120,124].

Bacterial pigments may exhibit lower stability levels than their synthetic counterparts since they are susceptible to various factors, including pH, temperature, UV radiation, oxygen, and heat, which can result in colour degradation and reduce shelf life [125]. Regulatory approval of bacterial pigments also hinges on meeting stringent stability and standardisation requirements. Agencies such as the FDA and EFSA demand that these pigments consistently conform to defined compositional and performance standards, ensuring product reliability and safety. However, achieving this consistency can be difficult due to the natural batch-to-batch variability inherent in biologically derived compounds. Additionally, manufacturers must conduct thorough stability testing to demonstrate that pigments maintain their integrity and efficacy over time and under varying environmental conditions. This is particularly critical for bacterial pigments, some of which may degrade or lose functionality when exposed to heat, light, or pH fluctuations. Meeting these criteria is essential, not only for regulatory compliance but also for successful commercial application [120]. Additionally, their range of colour shades may be more limited compared to synthetic dyes [38].

Continuously developing environmentally sustainable and energy-efficient techniques for pigment extraction and purification remains a persistent challenge. However, methods such as ultrasound-assisted extraction and enzyme-assisted extraction, which are considered eco-friendly methods, have been successfully employed in the extraction of these compounds [121]. On the other hand, it is crucial to optimise and control conditions such as pH, temperature, agitation, aeration, and composition of the medium to facilitate the scale-up process [126]. Strain improvement can also contribute to higher pigment production since wild strains might need longer fermentation processes, as well as producing lower quality pigments [126].

Industries still lack awareness regarding the potential advantages of bacterial pigments. Synthetic alternatives are typically perceived as more economical and user-friendly, posing challenges for natural dyes, especially bacterial pigments, to compete effectively [38].

Some solutions to the challenges described would be improving pigment production and colour variety through genetic engineering or by supplying species with additives that promote pigment production [84]. For example, several nitrogen and carbon sources to stimulate prodigiosin production in S. marcescens were tested, and the results showed that using peanut powder as a carbon source resulted in six times the amount of pigment [127]. Optimising fermentation procedures and improving product recovery and purification methods are essential for enhancing yields and minimising costs. Kulkarni successfully enhanced pigment production in *Kocuria flava* by implementing an ultrasonic bath treatment during bacterial growth. The initial pigment concentration of 18.18 μg/mL increased to 86.33 μg/mL by applying this treatment [128].

To tackle regulatory concerns, additional investigation is essential, alongside efforts with all the stakeholders to secure consumer approval. Raising awareness within industries about the advantages of bacterial pigments, particularly through LCA studies, could also help overcome these challenges [25,105,106].

## 9. Discussion and Future Perspectives

As we look towards the future, it becomes increasingly evident that bacterial pigments hold immense potential to reshape various fields and industries, especially textiles, and of all the existing microorganisms, bacteria must be considered an inexhaustible source of new natural pigments [129]. In this final chapter dedicated to future perspectives, we anticipate the untapped opportunities and emerging trends that are poised to propel bacterial pigments into new realms of innovation and impact. From advancements in production technologies to novel applications driven by evolving consumer needs and sustainability practices, the future of bacterial pigments promises to change the way we see and use colour.

**Figure 11:**
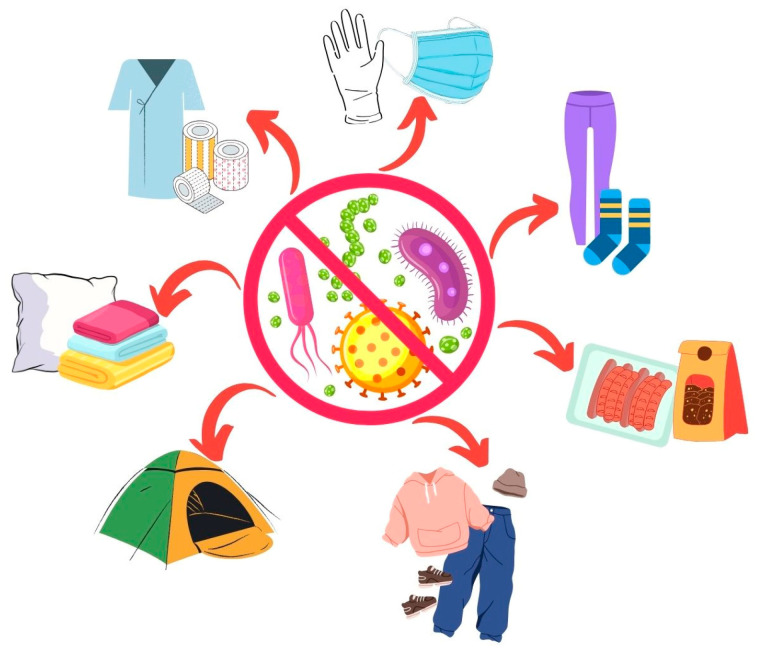
Possible antibacterial applications of bacterial pigments.

Bacterial pigments with antibacterial and antifungal properties have diverse applications across various textile sectors (Figure 11), enhancing both functionality and safety. In medical settings, these pigments can be applied to textiles such as hospital gowns, bed linen, and wound dressings to reduce the risk of healthcare-associated infections by inhibiting the growth of pathogenic bacteria and fungi. Similarly, personal protective equipment, including face masks, gloves, and protective clothing, can benefit from bacterial pigment-dyed textiles, providing an additional layer of defence against microbial contamination, especially in high-risk environments like healthcare facilities and laboratories.

In the sportswear and activewear industry, these antimicrobial properties can prevent the growth of odour-causing bacteria, maintaining freshness during prolonged use. Bacterial pigment-dyed textiles offer a natural and sustainable solution for odour control in athletic apparel, socks, and undergarments. Home textiles, such as bedding, towels, and upholstery, also benefit from these antimicrobial properties, reducing microbial contamination and enhancing hygiene. By minimising the growth of bacteria, mould, and mildew, these textiles can improve indoor air quality and prolong the lifespan of home furnishings.

Moreover, antimicrobial textiles can be incorporated into food packaging materials to inhibit spoilage microorganisms, extending the shelf life of perishable foods. Bacterial pigment-dyed textiles serve as effective antimicrobial packaging liners, wraps, and bags. In outdoor applications, such as tents, awnings, and outdoor furniture, these textiles help prevent mould, mildew, and algal growth, thereby enhancing the durability and longevity of outdoor fabrics. Finally, in the fashion industry, bacterial pigment-dyed textiles offer a unique selling proposition by combining antimicrobial functionality with aesthetic appeal in fashion-forward garments and accessories.

## Figures and Tables

**Table 1:** Bacterial pigments, antibacterial mechanisms, and which types of bacteria they are effective against.

Pigment	Antibacterial Mechanism	Effective Against	References
Pyocyanin	ROS generation	Gram-positive	[14,33,34]
Carotenoid	Outer membrane disruption ROS generation	Gram-negative	[19,20]
Melanin	Outer membrane disruption ROS generation Photothermal effect	Gram-positive and Gram-negative species	[25,26,27]
Prodigiosin	Outer membrane disruption Inhibition of cellular processes	Gram-positive species	[31,32,35]

**Table 2:** Bacterial pigments produced by each species and their fastness in textiles. Washing, perspiration, and rubbing fastness were rated on a scale of 1–6 using the grey scale; light fastness was rated on a scale of 1–8 using the blue wool scale.

Bacteria	Pigment	Substrates	Fastness
*Chryseobacterium* sp.	Carotenoid	Natural silk, Dubai silk, linen, Japanese cotton, and Indian cotton	No information
*Rhodonellum psychrophilum*	Carotenoid	Cotton, glace cotton, silk, and rayon	No information
*Metabacillus idrensis*	Carotenoid	Cotton	No information
*Streptomyces virginae*	Melanin	Wool	Perspiration: 4–5 Light: 5–6
*Streptomyces glaucescens*	Melanin	Cotton	Washing: 4–5
*Janthinobacterium lividum*	Violacein	Silk, cotton, wool, polyester, acrylic, acetate, vinylon, and nylon	
*Chromobacterium violaceum*	Violacein	Polyamide 6.6	Washing and perspiration: 5
*Chromobacterium violaceum*	Violacein	Pure cotton, pure silk, pure rayon, rayon jacquard, silk satin, cotton, and polyester	Silk: Light: 1 Washing: 2 Perspiration: 3–4 Other fibres: No information
	Prodigiosin		
*Vibrio* sp.		Wool, nylon, acrylic, and silk	No information
*Serratia* sp.	Prodigiosin	Cotton and wool	Washing: 4–5 and 3–4 Light: 4 and 2–3
*Serratia marcescens*	Prodigiosin	Wool, cotton, silk, nylon, and polyester	Pure silk: Light: 4–5 Washing: 4–5 Rubbing: 4–5 China silk: Light: 4–5
*Staphylococcus aureus*	Prodigiosin		Washing: 4–5 Rubbing: 4–5 Cotton: Washing: 4–5 Rubbing: 4–5
		Acrylic	No information
*Serratia rubidaea*	Prodigiosin	Cotton linen, cotton baft, cotton gabardine, cotton jersey, chiffon, satin, Dacron, and polyester	Satin and chiffon: ^1^ Light: 5 Washing: 6 Linen, baft, gabardine, Dacron: Light: 2 Washing: 6

^1^ The original results were converted to match the scale used in comparable studies to allow for correlation.

## Data Availability

No new data were created or analyzed in this study. Data sharing is not applicable to this article.

## Abbreviations

The following abbreviations are used in this manuscript:

AHL	acyl-homoserine
AL2(SO_4_)_3_	aluminium sulphate
Asm	acid sphingomyelinase
BBD	Box–Behnken design
BTCA	1,2,3,4-butanetetracarboxylic acid
Ca(OH)_2_	calcium hydroxide
COD	chemical oxygen demand
CO_2_	carbon dioxide
CrtB	phytoene synthase
CrtI	phytoene desaturase
CrtY	lycopene cyclase
CrtZ	hydroxylases
CuSO_4_	copper(II) sulphate
DAFS	dyeing after fermentation and sonication
DD	direct dyeing
EAE	enzyme-assisted extraction
FBR	fluidised bed reactor
FeSO_4_	ferrous sulphate
FTIR	Fourier-transform infrared spectroscopy
GGDPS	GGPP synthase
GGPP	geranylgeranyl diphosphate
H_2_O_2_	hydrogen peroxide
HPLC	high-performance liquid chromatography
HPH	high-pressure homogenisation
IL	ionic liquid
L-DOPA	L-3,4-dihydroxyphenylalanine
LB	Luria–Bertani
LCA	life cycle assessment
MAP	Methyl-3-n-Amylpyrrole
MBC	4-Methoxy-2,2′-Bipyrrole-5-Carbaldehyde
MEP	2-C-methyl-D-erythritol-4-phosphate
MVA	mevalonate
NaCl	sodium chloride
NaHCO_3_	sodium bicarbonate
O_2_·-	superoxide anions
PMS	paper mill sludge
PQS	Pseudomonas quinolone signal
ROS	reactive oxygen species
SFD	simultaneous fermentation and dyeing
SFE	supercritical fluid extraction
SM2	soybean meal
TBAOH	tetrabutylammonium hydroxide
TLC	thin-layer chromatography
UAE	ultrasound-assisted extraction
Y_2_O_3_	yttrium(III) oxide
ZrO_2_	zicornium dioxide
